# Patient satisfaction and patient-reported outcome measures after primary ankle arthrodesis: 2-year results from the Swedish Ankle Registry

**DOI:** 10.2340/17453674.2026.45374

**Published:** 2026-03-05

**Authors:** Alexandra UNDÉN, Lars JEHPSSON, Anders HENRICSON, Magnus K KARLSSON, Björn E ROSENGREN

**Affiliations:** 1 Department of Orthopedics and Clinical Sciences, Lund University and Skane University Hospital, Malmö; 2 Department of Radiology and Translational Sciences, Lund University and Skane University Hospital Malmö; 3 Department of Orthopedics, Falu Central Hospital and Center of Clinical Research Dalarna, Falun, Sweden

## Abstract

**Background and purpose:**

Ankle arthrodesis (AA) and total ankle replacement are the 2 main surgical treatment options for advanced ankle arthritis but information is scarce on satisfaction and patient-reported outcome measures (PROMs) after surgery. We aimed to evaluate postoperative satisfaction, changes in PROMs from before to 2 years after AA, and factors associated with the outcome.

**Methods:**

This is a national registry-based observational study using the Swedish Ankle Registry. We identified 1,145 patients who underwent AA between 2008 and 2020 who answered a question on satisfaction, and between 662 and 702 patients who answered pre- and 2-year postoperative PROMs (SElf reported Foot and Ankle Score [SEFAS], EQ-5D index, and EQ-5D VAS). We analyzed changes in scores and associations between surgical/patient factors (including preoperative PROMs) and the outcomes satisfaction/postoperative PROM score.

**Results:**

All mean PROM scores improved from before to 2 years postoperatively (all P < 0.001). 69% of the patients were satisfied with the procedure, 15% uncertain, and 16% dissatisfied. Satisfaction was associated with higher preoperative SEFAS score and dissatisfaction with lower preoperative SEFAS score. Higher SEFAS score was associated with male sex and osteoarthritis (compared with rheumatoid arthritis), while higher EQ-5D index and EQ-5D VAS scores were associated with male sex, posttraumatic arthritis, osteoarthritis (compared with rheumatoid arthritis), and higher age.

**Conclusion:**

The majority (69%) of patients treated with AA are satisfied with the procedure and a higher preoperative SEFAS score is associated with postoperative satisfaction. We speculate that preoperative SEFAS score may be useful for predicting postoperative outcomes and will facilitate preoperative patient discussions on expected results.

Ankle arthrodesis (AA) and total ankle replacement (TAR) represent the 2 most common surgical treatment methods for advanced ankle osteoarthritis. The only randomized controlled trial (RCT) comparing TAR and AA contributed with important but limited information [1] and choice of procedure largely remains in the hands of the surgeon and patient.

Many studies have shown adequate results after AA regarding healing and complications [2,3]. Although some smaller studies have pointed to adequate patient satisfaction after AA [4-6] others indicated that a substantial proportion of patients remain dissatisfied [7,8].

Some previous reports have also found that the outcome of AA may be influenced by sex and the preoperative disability, i.e., an association between preoperative patient-reported outcome measure (PROM) scores and postoperative improvement [9], and others that women have worse preoperative and postoperative pain and disability than men [10]. This implies that preoperative PROM scores may possibly be used as predictors of subjective outcome in patients with ankle arthritis [9] and that AA outcome studies should take sex into account [10].

We aimed to evaluate (i) patient satisfaction 2 years after AA, (ii) changes in PROMs from before to 2 years after AA, and (iii) factors associated with a better or worse outcome.

## Methods

This is a national registry-based observational study using data from the official national quality registry for ankle surgery (the Swedish Ankle Registry); the current coverage is 95% and completeness is 95%.

The Swedish Ankle Registry [11] contains information on AAs performed in Sweden since 1998. Patient data regarding sex, age, diagnosis, details on surgery, complications, and revision surgery are registered by the surgeon. Since 2008, generic and region-specific PROMs have been included in the registry and filled out by patients pre- and postoperatively, including a question on satisfaction with the surgery.

The study is reported according to STROBE guidelines.

### Outcomes

The generic EQ-5D includes an index (UK tariff) that estimates general health on a scale from –0.56 to best possible 1, as well as a visual analogue scale EQ-5D VAS for self-estimated general health status (0 to best possible 100). Foot and ankle function is evaluated by the Swedish validated region-specific SElf-reported Foot and Ankle Score (SEFAS) [12,13]. The SEFAS consists of 12 questions with 5 response options, each with a possible score of 0 to 4, with a total score of worst 0 to best possible 48. The performance of SEFAS is at least similar compared with the American Orthopaedic Foot and Ankle Society (AOFAS) score [14] and the Manchester–Oxford Foot Questionnaire (MOXFQ) [13].

We calculated score changes (Δ) by subtracting the preoperative scores from the postoperative scores. Minimal clinically important difference (MCID) is defined as the smallest difference in a score that is considered to be worthwhile or important [15]. For SEFAS MCID is 5 [16] for foot and ankle procedures.

There is no specific EQ-5D MCID value for surgical procedures of the ankle joint, and we used EQ-5D index 0.06 and EQ-5D VAS 11 according to Strand et al. [15]. Postoperatively, patients also answer a question on satisfaction with the surgical result on a 5-grade Likert scale (very satisfied, satisfied, neither satisfied nor dissatisfied, dissatisfied, or very dissatisfied).

### Statistics

Descriptive statistics were used to present the data, i.e., absolute frequencies, proportions (%) or means with standard deviations (SD). For inferential statistics we used means with 95% confidence intervals (CI). To analyze changes in PROMs we used paired samples t-tests. Due to the study design, floor and ceiling effects as well as regression to the mean may be present. Random slopes were tested to address possible floor and ceiling effects, but model fit did not improve. Any regression to the mean would likely have reduced rather than produced the observed associations and is unlikely to have affected the overall findings.

Satisfaction with surgery was recoded from the original 5-grade Likert scale into a 3-grade scale: “satisfied” (satisfied and very satisfied), “uncertain” (neither satisfied nor dissatisfied), and “dissatisfied” (dissatisfied and very dissatisfied). Based on this 3-grade scale, we created 2 separate dichotomous variables used in the logistic regression. In the first variable uncertain patients were grouped with dissatisfied patients to assess factors related to being satisfied vs being dissatisfied/uncertain. In the second variable uncertain patients were grouped with satisfied patients to assess factors associated with being dissatisfied vs being satisfied/uncertain. Henceforth in the text we will, if not otherwise specified, use the respective dichotomous variables when referring to satisfied and dissatisfied patients.

Our interest lies in analyzing associations between important available factors (preoperative PROM scores, sex, age, diagnosis, and procedure) and the outcomes: satisfaction and postoperative PROM score. Previous publications indicate that sex, age, diagnosis, and preoperative PROM score are associated with postoperative PROM score [4,9,17], and we suspected that this applied to satisfaction as well. Sex, age, and diagnosis may also influence the exposure (i.e., surgery) and we therefore, where applicable, adjusted for these factors.

As a considerable number of patients had only 1 observation (pre- or postoperative) (Figure), we utilized multiple imputation (5 iterations) to impute pre- or postoperative data using age, sex, diagnosis, procedure, existing pre- and postoperative PROMs, and satisfaction/dissatisfaction as predictors. We then performed logistic regression models on the imputed data to analyze associations between satisfaction/dissatisfaction and preoperative PROMs, age, sex, diagnosis, and procedure. We included patients who answered a pre- or postoperative PROM, as well as the question on satisfaction (n = 1,117). We performed 2 analyses per PROM (6 in total), adjusting for age, sex, procedure, and diagnosis in all models, as well as for the preoperative PROM being investigated. Outcomes were either satisfaction or dissatisfaction. Pseudo R squared (Nagelkerke) is provided as an indicator of goodness of fit.

**Figure:**
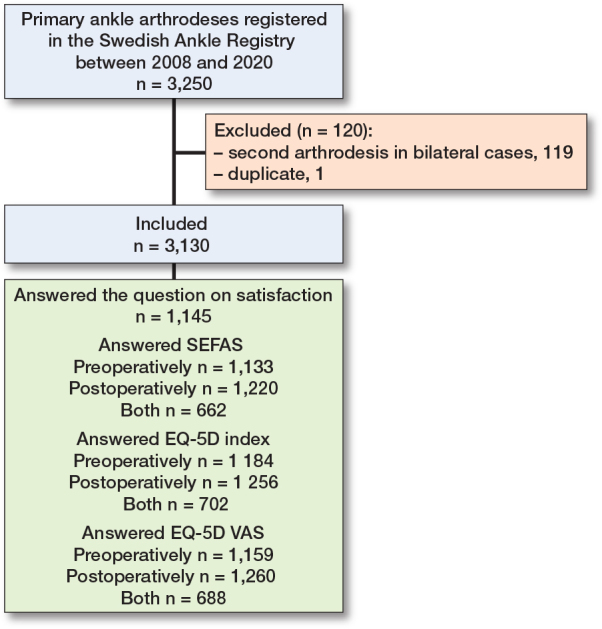
Flowchart of patient selection and participation, showing the 3,250 primary ankle arthrodeses (AA) registered in the Swedish Ankle Registry between 2008 and 2020.

We used mixed-effect models to examine the associations between the 3 PROMs (outcome) and age, sex, and diagnosis. 3 mixed-effects models were fitted (1 for each PROM), each with a subject-specific random intercept and no additional random effects. Models were adjusted for age, sex, diagnosis, timepoint, and a sex-by-timepoint interaction. Model adequacy was assessed through residual diagnostics, including residuals vs predicted values and normal probability plots, with no major violations of assumptions observed.

To build more robust statistical models, rare diagnoses such as septic arthritis, pes equinovarus deformity, and diabetic enthesopathy were added to “Other” diagnoses. The 4 most common procedures—“open screw fixation,” “arthroscopic screw fixation,” intramedullary nail,” and “plate”—were included, excluding less common procedures such as external fixation and staples as well as missing data (total n = 111/3,130) from the logistic regression analysis. For reference we provide results from complete case analyses in Supplementary Tables 1 and 2. We used the Statistical Package for the Social Sciences (SPSS) version 26 (IBM Corp, Armonk, NY, USA) for statistical analyses.

### Ethics, funding, and disclosures

The study was approved by the Ethical Review Board of Lund University (Dnr 2014/448) and conducted in accordance with the Helsinki Protocol. The work was supported by grants from ALF and the FoUU of Region Skåne, Greta Koch, Herman Järnhardt, Maggie Stephens, Guldbyxan, and Skåne University Hospital foundations. Funders had no influence on the design of the study, the collection, analysis, or interpretation of data, on writing the manuscript, or in any other part of the study.

The authors declare no conflicts of interest. Complete disclosure of interest forms according to ICMJE are available on the article page, doi: 10.2340/17453674.2026.45374

## Results

Between 2008 and 2020, we identified 3,250 patients in the Swedish Ankle Registry who had undergone primary AA (Figure). There were 119 bilateral cases and 1 duplicate. The duplicate was excluded and to avoid selection bias we excluded the second arthrodesis in the bilateral cases, leaving 3,130. Of these, 55% were male, mean age was 62 (SD 13), and distribution of diagnoses was posttraumatic arthritis (PtA) 44%, osteoarthritis (OA) 29%, rheumatoid arthritis (RA) 10%, other 13%, diabetic enthesopathy 2%, septic arthritis 1%, and pes equinovarus 1%. Data on included patients and non-responders regarding preoperative PROMs, age, sex, and diagnosis are presented in Table 1.

**Table 1:** Demographics of patients with and without pre- and postoperative data

Factor	With data	Without data
Preoperative SEFAS		
Patients, n	1,133	1,997
Men (%)	58	54
Age (SD)	63 (13)	61 (13)
Diagnosis (%)		
RA	10	11
PtA	43	44
OA	32	27
Other	15	18
Preoperative EQ-5D index		
Patients, n	1,184	1,946
Men (%)	58	53
Age (SD)	63 (13)	61 (13)
Diagnosis (%)		
RA	9	11
PtA	43	44
OA	32	27
Other	16	18
Preoperative EQ-5D VAS		
Patients, n	1,159	1,971
Men (%)	57	54
Age (SD)	62 (13)	62 (13)
Diagnosis (%)		
RA	9	11
PtA	43	44
OA	32	27
Other	16	18
Information on satisfaction		
Patients, n	1,145	1,985
Men (%)	58	54
Age (SD)	64 (12)	61 (14)
Diagnosis (%)		
RA	10	10
PtA	39	46
OA	35	35
Other	15	18
Preoperative values		
SEFAS **^a^**	16 (6)	15 (7)
EQ-5D index **^a^**	0.37 (0.32)	0.33 (0.32)
EQ-5D VAS **^a^**	55 (22)	54 (22)

OA: osteoarthritis, PtA: posttraumatic arthritis, RA: rheumatoid arthritis, SEFAS: SElf-reported Foot and Ankle Score.

a Values are mean (SD) and number of patients are as stated under preoperative SEFAS, EQ-5D index, and EQ-VAS.

2 years after AA surgery 1,145 patients (37%) answered the question on satisfaction (see Figure).

### Outcomes

69% of responders were satisfied, 15% uncertain, and 16% dissatisfied. All mean PROM scores improved from pre- to 2 years postoperatively (all P < 0.001) and all mean changes were clinically relevant (> MCID values) (Table 2).

**Table 2:** Mean changes (Δ scores) of patient-reported outcome measure (PROM) scores from preoperatively to 2 years postoperatively with 95% confidence interval (CI)

PROM	MCID	n	Δ score (CI)	P value
SEFAS	5	662	15 (14–16)	< 0.001
EQ-5D index	0.06	702	0.32 (0.30–0.35)	< 0.001
EQ-5D VAS	11	688	14 (12–16)	< 0.001

MCID: minimal clinically important difference. SEFAS: SElf-reported Foot and Ankle Score.

We found that a higher preoperative SEFAS score was associated with satisfaction (Table 3) and also that a lower preoperative SEFAS score was associated with dissatisfaction (Table 4). Furthermore, those with the diagnosis OA (compared with RA), were more likely to be satisfied (Table 3). We found no apparent associations between procedure, preoperative EQ-5D index, or preoperative EQ-5D VAS with either satisfaction or dissatisfaction (Tables 3 and 4).

**Table 3:** Associations between satisfaction 2 years postoperatively and preoperative PROM, age, sex, diagnosis, and procedure. Logistic regression on imputed data with 95% confidence interval (CI)

Variable	Odds ratio for satisfaction (CI) Imputed data with preoperative		
	SEFAS n = 1,117	EQ-5D index n = 1,117	EQ-5D VAS n = 1,117
Preoperative PROM	1.04 (1.01–1.07)	1.38 (0.79–2.42)	1.01 (1.00–1.01)
Age	0.99 (0.98–1.01)	0.99 (0.98–1.01)	1.00 (0.98–1.01)
Sex			
Female	Reference	Reference	Reference
Male	1.03 (0.78–1.35)	1.07 (0.81–1.40)	1.07 (0.82–1.41)
Diagnosis			
RA	Reference	Reference	Reference
PtA	1.05 (0.66–1.67)	1.07 (0.68–1.70)	1.06 (0.67 1.68)
OA	1.72 (1.06–2.80)	1.75 (1.08–2.83)	1.72 (1.06–2.79)
Other	0.75 (0.44–1.25)	0.81 (0.49–1.35)	0.82 (0.49–1.35)
Procedure			
Open screw	Reference	Reference	Reference
Plate	0.80 (0.54–1.20)	0.82 (0.55–1.22)	0.81 (0.54–1.21)
Intramedullary nail	1.13 (0.79–1.62)	1.11 (0.77–1.59)	1.10 (0.77–1.58)
Arthroscopic screw	1.13 (0.78–1.66)	1.18 (0.81–1.72)	1.17 (0.80–1.71)
Pseudo R squared (Nagelkerke)	0.042	0.029	0.031

For abbreviations, see Table 1.

**Table 4:** Associations between dissatisfaction 2 years postoperatively and preoperative PROM, age, sex, diagnosis, and procedure. Logistic regression on imputed data with 95% confidence interval (CI)

Variable	Odds ratio for dissatisfaction (CI) Imputed data with preoperative		
	SEFAS n = 1,117	EQ-5D index n = 1,117	EQ-5D VAS n = 1,117
Preoperative PROM	0.96 (0.93–0.99)	0.99 (0.38–2.55)	1.00 (0.98–1.01)
Age	0.99 (0.98–1.01)	0.99 (0.98–1.01)	0.99 (0.98–1.01)
Sex			
Female	Reference	Reference	Reference
Male	1.14 (0.81–1.60)	0.07 (0.76–1.49)	1.08 (0.77–1.51)
Diagnosis			
RA	Reference	Reference	Reference
PtA	0.86 (0.49–1.51)	0.83 (0.47–1.45)	0.86 (0.49–1.51)
OA	0.66 (0.37–1.19)	0.64 (0.36–1.15)	0.66 (0.37–1.19)
Other	1.09 (0.59–2.03)	0.99 (0.54–1.83)	1.02 (0.55–1.87)
Procedure			
Open screw	Reference	Reference	Reference
Plate	1.15 (0.70–1.89)	1.17 (0.72–1.91)	1.14 (0.70–1.86)
Intramedullary nail	0.99 (0.64–1.53)	1.01 80.65–1.56)	1.02 (0.66–1.57)
Arthroscopic screw	0.88 (0.55–1.42)	0.86 (0.53–1.37)	0.86 (0.54–1.38)
Pseudo R squared (Nagelkerke)	0.030	0.014	0.016

For abbreviations, see Table 1.

In the mixed-effects model, we found that male sex and diagnoses OA and “other” (compared with RA) were associated with higher SEFAS score. Male sex, diagnoses PtA and OA (compared with RA), as well as higher age, were associated with higher EQ-5D index score. Male sex, diagnoses PtA, OA and “other” (compared with RA), as well as higher age, were associated with higher EQ-5D VAS (Table 5).

**Table 5:** Associations between PROMS and age, sex, and diagnosis

	SEFAS Mixed model coefficient (CI) n = 1,749/6,260	EQ-5D index Mixed model coefficient (CI) n = 1,840/6,260	EQ-5D VAS Mixed model coefficient (CI) n = 1,803/6,260
Age	0.03 (–0.003 to 0.05)	0.003 (0.002 to 0.004)	0.09 (0.02 to 0.17)
Sex			
Female	Reference	Reference	Reference
Male	1.71 (0.94 to 2.47)	0.08 (0.04 to 0.11)	3.29 (0.74 to 5.83)
Diagnosis			
RA	Reference	Reference	Reference
PtA	1.02 (–0.21 to 2.24)	0.08 (0.03 to 0.12)	8.46 (5.17 to 11.8)
OA	1.35 (0.09 to 2.61)	0.09 (0.04 to 0.14)	9.27 (5.87 to 12.7)
Other	1.56 (0.16 to 2.96)	0.01 (–0.04 to 0.07)	5.56 (1.82 to 9.30)
Timepoint			
Primary	Reference	Reference	Reference
Secondary	13.8 (12.8 to 14.8)	0.35 (0.32 to 0.38)	14.4 (12.1 to 16.8)
Sex/timepoint interaction	1.36 (0.04 to 2.69)	–0.03 (–0.07 to 0.02)	–0.52 (–3.62 to 2.57)

For abbreviations, see Table 1.

## Discussion

We aimed to evaluate postoperative satisfaction, changes in PROMs from before to 2 years after AA and factors associated with a better/worse outcome.

We found that 69% of patients were satisfied with the AA, and that all mean PROM scores had improved statistically and clinically significantly 2 years after surgery. Higher preoperative SEFAS score and the diagnosis OA were associated with satisfaction, while a lower preoperative SEFAS score was associated with dissatisfaction. Male sex, some diagnoses, and higher age were associated with higher PROMs.

Although the definition of satisfaction and follow-up times varies, our results on satisfaction, 69% 2 years after AA surgery, are comparable to 75% satisfaction 2 years after AA in 88 patients in Sudan [6] and 69% satisfaction mean 5.5 years after AA and contralateral TAR in 16 patients in Sweden [18]. Our results were better than 47% satisfaction at mean 2 years after salvage AA in 68 patients in Sweden [19], but worse than 89% at mean 4 years after bilateral AA in 35 patients in Sweden [4]. Our results were also similar to 72% satisfaction 2 years after TAR in 126 patients in Sweden [20].

We found that higher preoperative SEFAS was associated with satisfaction and lower preoperative SEFAS was associated with dissatisfaction. These findings indicate that preoperative PROMs, at least the region-specific SEFAS, could be a predictor of postoperative outcome in terms of PROM score, supported by the findings of Waly et al. who found an association between preoperative PROMs (Ankle Osteoarthritis Scale and Ankle Arthritis Score) and achieving MCID in physical and mental function [9]. However, our findings also indicate that preoperative SEFAS could facilitate prediction of postoperative satisfaction/dissatisfaction. This could further aid in the preoperative discussion with the patient on available treatments and expected results after AA. The fact that we were unable to find an association between satisfaction and preoperative EQ-5D index or EQ-5D VAS could partly be explained by the fact that the EQ-5D score reflects all aspects of the patient’s general health, as opposed to the specific question of satisfaction with the operated-on ankle, and could thus be expected to be less responsive.

We were unable to find an association between sex and satisfaction/dissatisfaction in the adjusted analyses, despite the fact that we, and others, have found that men score better than women on some PROMs including SEFAS [21] and EQ-5D index [22]. Male sex was associated with higher PROMs (SEFAS, EQ-5D index, and EQ-5D VAS), in line with previous reports, which have shown that men score better on PROMs than women [9,21-23]. We found that RA was associated with lower EQ-5D index and EQ-5D VAS compared with PtA and OA, and with lower SEFAS compared with OA. This could be expected with a systemic disease, and has been found in earlier studies [4,20]. We found that higher age was associated with higher EQ-5D index and EQ-5D VAS scores, consistent with the association between preoperative EQ-5D and higher age reported 2 years after TAR in 126 Swedish patients [20], but contrary to the pattern observed in the general Swedish population [22].

### Strengths

The strengths of the current study include the large patient cohort and that the data reflects both the Swedish population and the national Swedish healthcare system, with many different caregivers and surgeons of varying experience, in contrast to reports from single or specialized units or surgeons. Another strength is the combination of generic (EQ-5D) and region-specific (SEFAS) PROMs.

### Limitations

Limitations include a risk of selection bias, low participation, incomplete reporting, and data only from Sweden. Although the dropout table (Table 1) did not indicate any major differences, excluded patients were younger and diagnosis, as well as sex, was distributed differently. In order to build more robust statistical models, rare AA procedures were excluded from the regression models, precluding generalizability to groups undergoing these procedures. Another limitation is lack of detailed information on non-responders; trends towards poorer subjective results from this group have been described after other orthopedic procedures [24,25]. With these limitations in mind, generalizability may be questioned.

Confounding by indication is plausible, in that choice of method (AA or TAR) and surgical approach may have been influenced by both preference of the surgeon as well as specific patient factors, none of which we were able to take into account. Although validated for several languages, the SEFAS score has been used in only a few other studies related specifically to AA [4,18,19], which impedes putting results into perspective. Another limitation is that information on patients with severe end-stage OA who do not undergo surgery is missing in our study and within the field.

Our results cover only the 2-year postoperative period. Studies with longer or much longer follow-up are scarce and most often small [2,4,8,9,18]. Some have, however, indicated that AA may be associated with not so favorable outcomes in the long-term perspective, at least regarding degeneration of adjacent joints, but more studies have been deemed necessary [1,2].

### Conclusion

Our study indicates that most patients treated with AA who responded to the questionnaire were satisfied with the surgery, and that a high preoperative SEFAS score is associated with satisfaction after surgery.

*In perspective*, AA seems a reasonable, but not optimal, treatment for end-stage ankle arthritis and we speculate that preoperative SEFAS score may be useful for predicting surgical outcome and facilitate preoperative patient discussions on expected results. Long-term studies on PROMs and satisfaction after AA are needed.

### Supplementary data

Supplementary Tables 1–2 are available as Supplementary data on the article page, doi: 10.2340/17453674.2026.45374

## Supplementary Material



## Notes

AU, LJ, AH, MK and BR designed the study; AU and BR collected data; AU, LJ, and BR interpreted data and did statistical analyses; AU and BR wrote the first version, all authors finalized the manuscript together.

Handling co-editors: Jeppe Vejlgaard Rasmussen and Philippe Wagner

*Acta* thanks Helka Koivu and Dawson Muir for help with peer review of this manuscript.

## References

[1] Goldberg A J, Chowdhury K, Bordea E, Hauptmannova I, Blackstone J, Brooking D, et al. Total ankle replacement versus arthrodesis for end-stage ankle osteoarthritis: a randomized controlled trial. Ann Intern Med 2022; 175: 1648-57. doi: 10.7326/M22-2058. 36375147

[2] Ferguson Z, Anugraha A, Janghir N, Pillai A. Ankle arthrodesis: a long term review of the literature. J Orthop 2019; 16: 430-3. doi: 10.1016/j.jor.2019.08.004. 31496552 PMC6722282

[3] Adukia V, Mangwani J, Issac R, Hussain S, Parker L. Current concepts in the management of ankle arthritis. J Clin Orthop Trauma 2020; 11: 388-98. doi: 10.1016/j.jcot.2020.03.020. 32405197 PMC7211821

[4] Henricson A, Kamrad I, Rosengren B, Carlsson A. Bilateral arthrodesis of the ankle joint: self-reported outcomes in 35 patients from the Swedish Ankle Registry. J Foot Ankle Surg 2016; 55: 1195-8. doi: 10.1053/j.jfas.2016.07.014. 27614825

[5] Hendrickx R P, Stufkens S A, de Bruijn E E, Sierevelt I N, van Dijk C N, Kerkhoffs G M. Medium- to long-term outcome of ankle arthrodesis. Foot Ankle Int 2011; 32: 940-7. doi: 10.3113/FAI.2011.0940. 22224322

[6] Nogod S, Khairy A M M Jr, Nubi O G, Fatooh M S, Mohammed Ali Abd-Elmaged H. Ankle arthrodesis: indications, outcomes, and patient satisfaction. Cureus 2023; 15: e37177. doi: 10.7759/cureus.37177. 37034142 PMC10076242

[7] Younger A S, Wing K J, Glazebrook M, Daniels T R, Dryden P J, Lalonde K A, et al. Patient expectation and satisfaction as measures of operative outcome in end-stage ankle arthritis: a prospective cohort study of total ankle replacement versus ankle fusion. Foot Ankle Int 2015; 36: 12334. doi: 10.1177/1071100714565902. 25645533

[8] Coester L M, Saltzman C L, Leupold J, Pontarelli W. Long-term results following ankle arthrodesis for post-traumatic arthritis. J Bone Joint Surg Am 2001; 83: 219-28. doi: 10.2106/00004623-200102000-00009. 11216683

[9] Waly F J, Yeo E M N, Wing K J, Penner M J, Veljkovic A, Younger A S E. Relationship of preoperative patient-reported outcome measures (PROMs) to postoperative success in end-stage ankle arthritis. Foot Ankle Int 2020; 41: 253-8. doi: 10.1177/1071100719893334. 32045278

[10] Dodd A, Pinsker E, Younger A S E, Penner M J, Wing K J, Dryden P J, et al. Sex differences in end-stage ankle arthritis and following total ankle replacement or ankle arthrodesis. J Bone Joint Surg Am 2022; 104: 221-8. doi: 10.2106/JBJS.21.00287. 35007215

[11] Swedish Ankle Registry. Annual report 2024. Available from: http://www.swedankle.se

[12] Coster M C, Bremander A, Rosengren B E, Magnusson H, Carlsson A, Karlsson M K. Validity, reliability, and responsiveness of the Self-reported Foot and Ankle Score (SEFAS) in forefoot, hindfoot, and ankle disorders. Acta Orthop 2014; 85: 187-94. doi: 10.3109/17453674.2014.889979. 24564747 PMC3967263

[13] Arbab D, Kuhlmann K, Schnurr C, Luring C, Konig D, Bouillon B. Comparison of the Manchester–Oxford Foot Questionnaire (MOXFQ) and the Self-Reported Foot and Ankle Outcome Score (SEFAS) in patients with foot or ankle surgery. Foot Ankle Surg 2019; 25: 361-5. doi: 10.1016/j.fas.2018.01.003. 30321978

[14] Coster M C, Rosengren B E, Bremander A, Brudin L, Karlsson M K. Comparison of the Self-reported Foot and Ankle Score (SEFAS) and the American Orthopedic Foot and Ankle Society Score (AOFAS). Foot Ankle Int 2014; 35: 1031-6. doi: 10.1177/1071100714543647. 25015390

[15] Strand V, Boers M, Idzerda L, Kirwan J R, Kvien T K, Tugwell P S, et al. It’s good to feel better but it’s better to feel good and even better to feel good as soon as possible for as long as possible. Response criteria and the importance of change at OMERACT 10. J Rheumatol 2011; 38: 1720-7. doi: 10.3899/jrheum.110392. 21807792

[16] Coster M C, Nilsdotter A, Brudin L, Bremander A. Minimally important change, measurement error, and responsiveness for the Self-Reported Foot and Ankle Score. Acta Orthop 2017; 88: 300-4. doi: 10.1080/17453674.2017.1293445. 28464751 PMC5434599

[17] Rajapakshe S, Sutherland J M, Wing K, Crump T, Liu G, Penner M, et al. Health and quality of life outcomes among patients undergoing surgery for end-stage ankle arthritis. Foot Ankle Int 2019; 40: 1129-39. doi: 10.1177/1071100719856888. 31215232

[18] Henricson A, Fredriksson M, Carlsson A. Total ankle replacement and contralateral ankle arthrodesis in 16 patients from the Swedish Ankle Registry: Self-reported function and satisfaction. Foot Ankle Surg 2016; 22: 32-4. doi: 10.1016/j.fas.2015.04.007. 26869497

[19] Kamrad I, Henricson A, Magnusson H, Carlsson A, Rosengren B E. Outcome After salvage arthrodesis for failed total ankle replacement. Foot Ankle Int 2016; 37: 255-61. doi: 10.1177/1071100715617508. 26582180

[20] Kamrad I, Carlsson A, Henricson A, Magnusson H, Karlsson M K, Rosengren B E. Good outcome scores and high satisfaction rate after primary total ankle replacement. Acta Orthop 2017; 88: 675-80. doi: 10.1080/17453674.2017.1366405. 28812410 PMC5694814

[21] Coster M C, Rosengren B E, Karlsson M K, Carlsson A. Age- and gender-specific normative values for the Self-Reported Foot and Ankle Score (SEFAS). Foot Ankle Int 2018; 39: 1328-34. doi: 10.1177/1071100718788499. 30035614

[22] Burstrom K, Johannesson M, Diderichsen F. Swedish population health-related quality of life results using the EQ-5D. Qual Life Res 2001; 10: 621-35. doi: 10.1023/a:1013171831202. 11822795

[23] Teni F S, Rolfson O, Devlin N, Parkin D, Naucler E, Burstrom K, et al. Longitudinal study of patients’ health-related quality of life using EQ-5D-3L in 11 Swedish National Quality Registers. BMJ Open 2022; 12: e048176. doi: 10.1136/bmjopen-2020-048176. PMC8739074 34992101

[24] Kim J, Lonner J H, Nelson C L, Lotke P A. Response bias: effect on outcomes evaluation by mail surveys after total knee arthroplasty. J Bone Joint Surg Am 2004; 86: 15-21. 14711940

[25] Zini M L L, Banfi G. A narrative literature review of bias in collecting patient reported outcomes measures (PROMs). Int J Environ Res Public Health 2021; 18. doi: 10.3390/ijerph182312445. 34886170 PMC8656687

