# Supplementary material for: Patient satisfaction and patient-reported outcome measures after primary ankle arthrodesis: 2-year results from the Swedish Ankle Registry
Source: Acta Orthop. 2026 Mar 5;97:171–6. doi: 10.2340/17453674.2026.45374 (PMC12963958; doi:10.2340/17453674.2026.45374)
Supplement: Supplementary file 1 [file ActaO-97-45374-s1.pdf]

SUPPLEMENTARY DATA

Supplementary Table 1A. Associations between satisfaction 2 years postoperatively and preoperative PROM, age, sex, diagnosis, and procedure. Complete case analysis, Logistic regression

	Complete case analysis with preoperative SEFAS	Complete case analysis with preoperative EQ-5D index	Complete case analysis with preoperative EQ-5D VAS
Variable	Odds ratio for satisfaction (CI) n = 593	Odds ratio for satisfaction (CI) n = 618	Odds ratio for satisfaction (CI) n = 607
Preoperative PROM	1.04 (1.00–1.07)	1.38 (0.79–2.44)	1.01 (1.00–1.01)
Age	0.99 (0.97–1.01)	0.99 (0.98–1.01)	0.99 (0.97–1.01)
Sex			
Female	Reference	Reference	Reference
Male	1.46 (0.99–2.15)	1.47 (1.01–2.14)	1.50 (1.01–2.19)
Diagnosis			
RA	Reference	Reference	Reference
PtA	1.13 (0.60–2.13)	0.95 (0.50–1.81)	0.95 (0.49–1.85)
OA	1.50 (0.77–2.91)	1.32 (0.67–2.58)	1.31 (0.66–2.59)
Other	0.83 (0.40–1.70)	0.71 (0.35–1.45)	0.73 (0.35–1.50)
Procedure			
Open screw	Reference	Reference	Reference
Plate	0.73 (0.40–1.36)	0.65 (0.36–1.15)	0.58 (0.33–1.02)
Intramedullary nail	1.16 (0.70–1.94)	1.04 (0.64–1.68)	1.09 (0.65–1.80)
Arthroscopic screw	1.67 (0.98–2.84)	1.74 (1.03–2.95)	1.53 (0.91–2.58)
Pseudo R squared (Nagelkerke)	0.055	0.047	0.046

For abbreviations, see Table 2.

Supplementary Table 1B. Associations between dissatisfaction 2 years postoperatively and preoperative PROM, age, sex, diagnosis, and procedure. Complete case analysis, Logistic regression

	Complete case analysis with preoperative SEFAS	Complete case analysis with preoperative EQ-5D index	Complete case analysis with preoperative EQ-5D VAS
Variable	Odds ratio for dissatisfaction (CI) n = 593	Odds ratio for dissatisfaction (CI) n = 618	Odds ratio for dissatisfaction (CI) n = 607
Preoperative PROM	0.96 (0.92–0.99)	0.93 (0.46–1.86)	0.99 (0.98–1.01)
Age	1.00 (0.98–1.02)	1.00 (0.98–1.02)	1.00 (0.98–1.02)
Sex			
Female	Reference	Reference	Reference
Male	0.80 (0.50–1.28)	0.73 (0.46–1.16)	0.71 (0.45–1.14)
Diagnosis			
RA	Reference	Reference	Reference

PtA	0.77 (0.37–1.61)	1.03 (0.48–2.25)	0.91 (0.42–1.95)
OA	0.59 (0.27–1.29)	0.78 (0.35–1.77)	0.71 (0.32–1.57)
Other	0.91 (0.39–2.11)	1.12 (0.47–2.64)	0.99 (0.43–2.31)
Procedure			
Open screw	Reference	Reference	Reference
Plate	1.57 (0.75–3.31)	1.85 (0.94–3.66)	1.93 (0.97–3.83)
Intramedullary nail	1.36 (0.75–2.48)	1.45 (0.81–2.60)	1.61 (0.89–2.92)
Arthroscopic screw	0.87 (0.45–1.67)	0.85 (0.45–1.62)	0.91 (0.48–1.74)
Pseudo R squared (Nagelkerke)	0.042	0.025	0.033

For abbreviations, see Table 1.

Supplementary Table 2. **Associations between pre- and postoperative PROMs with age, sex, diagnosis, and procedure**

Factor	Preoperative PROM coefficient (CI)	Postoperative PROM coefficient (CI)
SEFAS	n = 1,132	n = 643
Age	0.02 (–0.01 to 0.06)	0.05 (–0.02 to 0.12)
Sex		
Female	reference	reference
Male	1.84 (1.06 to 2.62)	3.00 (1.35 to 4.65)
Diagnosis		
RA	reference	reference
PtA	1.34 (–0.01 to 2.69)	–2.79 (–3.59 to 2.03)
OA	1.11 (–0.29 to 2.51)	1.32 (–1.57 to 4.22)
Other	2.69 (1.14 to 4.24)	–2.79 (–6.00 to 0.42)
Procedure		
Open screw	Reference	Reference
Plate	–	–3.10 (–5.86 to -0.33)
Intramedullary nail	–	–1.48 (–3.63 to 0.68)
Arthroscopic screw	–	0.81 (–1.31 to 2.92)
Preoperative PROM	–	0.46 (0.34 to 0.58)
EQ-5D index	n = 1,183	n = 683
Age	0.004 (0.003 to 0.01)	0.002 (< 0.001 to 0.004)
Sex		
Female	Reference	Reference
Male	0.08 (0.04 to 0.12)	0.04 (–0.004 to 0.09)
Diagnosis		
RA	Reference	Reference
PtA	0.10 (0.04 to 0.17)	0.05 (–0.03 to 0.12)
OA	0.08 (0.01 to 0.15)	0.10 (0.02 to 0.18)
Other	0.08 (0.001 to 0.15)	–0.03 (–0.12 to 0.06)
Procedure		
Open screw	–	Reference
Plate	–	–0.06 (–0.13 to 0.01)
Intramedullary nail	–	–0.03 (–0.09 to 0.03)
Arthroscopic screw	–	0.05 (–0.01 to 0.10)
Preoperative PROM	–	0.24 (0.17 to 0.30)
EQ-VAS	n = 1,158	n = 670

Age	0.15 (0.05 to 0.26)	-0.02 (–0.15 to 0.10)
Sex		
Female	Reference	Reference
Male	4.09 (1.48 to 6.70)	4.84 (1.77 to 7.91)
Diagnosis		
RA	Reference	Reference
PtA	7.47 (2.84 to 12.10)	6.23 (0.66 to 11.81)
OA	6.96 (2.16 to 11.75)	8.44 (2.75 to 14.14)
Other	5.28 (0.07 to 10.48)	5.04 (–1.11 to 11.81)
Procedure		
Open screw	–	Reference
Plate	–	–6.70 (–11.65 to –1.75)
Intramedullary nail	–	–4.30 (–8.42 to –0.17)
Arthroscopic screw	–	1.06 (–2.95 to 5.07)
Preoperative PROM	–	0.26 (0.19 to 0.33)

For abbreviations, see Table 1.
